# New molecular staging with G-factor supplements TNM classification in gastric cancer: a multicenter collaborative research by the Japan Society for Gastroenterological Carcinogenesis G-Project committee

**DOI:** 10.1007/s10120-014-0338-2

**Published:** 2014-02-01

**Authors:** Tetsuji Sawada, Masakazu Yashiro, Kazuhiro Sentani, Naohide Oue, Wataru Yasui, Kohji Miyazaki, Keita Kai, Sachio Fushida, Takashi Fujimura, Masaichi Ohira, Yoshihiro Kakeji, Shoji Natsugoe, Ken Shirabe, Sachiyo Nomura, Yutaka Shimada, Naohiro Tomita, Kosei Hirakawa, Yoshihiko Maehara

**Affiliations:** 1 Department of Surgical Oncology, Osaka City University Graduate School of Medicine, 1-4-3 Asahimachi, Abeno-ku, Osaka, 545-8585 Japan; 2 Oncology Institute of Geriatrics and Medical Science, Osaka City University Graduate School of Medicine, Osaka, Japan; 3 Department of Molecular Pathology, Hiroshima University Institute of Biomedical and Health Sciences, Hiroshima, Japan; 4 Department of Surgery, Saga University Faculty of Medicine, Saga, Japan; 5 Department of Pathology and Microbiology, Saga University Faculty of Medicine, Saga, Japan; 6 Division of Cancer Medicine, Department of Gastroenterologic Surgery, Graduate School of Medical Science, Kanazawa University, Kanazawa, Japan; 7 Division of Gastrointestinal Surgery, Department of Surgery, Graduate School of Medicine, Kobe University, Kobe, Japan; 8 Digestive Surgery Surgical Oncology, Kagoshima University School of Medicine, Kagoshima, Japan; 9 Department of Surgery and Science, Graduate School of Medical Sciences, Kyushu University, Fukuoka, Japan; 10 Department of Gastrointestinal Surgery, Graduate School of Medicine, University of Tokyo, Tokyo, Japan; 11 Department of Surgery and Science, Graduate School of Medicine and Pharmaceutical Sciences for Research, University of Toyama, Toyama, Japan; 12 Division of Lower GI, Department of Surgery, Hyogo College of Medicine, Hyogo, Japan

**Keywords:** Gastric cancer, G-factor, Molecular staging, TNM classification

## Abstract

**Background:**

The G-Project committee was erected by the Japan Society for Gastroenterological Carcinogenesis with an aim of establishing a new classification scheme based on molecular biological characteristics that would supplement the conventional TNM classification to better predict outcome.

**Methods:**

In a literature search involving 822 articles on gastric cancer, eight molecules including p53, vascular endothelial growth factor (VEGF)-A, VEGF-C, matrix metalloproteinase-7 (MMP-7), human epidermal growth factor receptor 2, Regenerating islet-derived family, member 4, olfactomedin-4 and Claudin-18 were selected as candidates to be included in the new molecular classification scheme named G-factor. A total of 210 cases of gastric cancer who underwent curative R0 resection were registered from four independent facilities. Immunohistochemical staining for the aforementioned molecules was performed for the surgically resected specimens of the 210 cases to investigate the correlation between clinicopathological factors and expression of each molecule.

**Results:**

No significant correlation was observed between the immunostaining expression of any of the eight factors and postoperative recurrence. However, the expressions of p53 and MMP-7 were significantly correlated with overall survival (OS). When 210 gastric cancer patients were divided into three groups based on the expression of p53 and MMP-7 (G0 group: negative for both p53 and MMP-7, *n* = 69, G1 group: positive for either p53 or MMP-7, *n* = 97, G2 group: positive for both of the molecules, *n* = 44), G2 group demonstrated significantly higher recurrence rate (59 %) compared to 38 % in G0 (*p* = 0.047). The multivariate regression analysis revealed that G2 group was independently associated with a shorter disease-free survival (DFS) (hazard ratio 1.904, 95 % CI 1.098–3.303; *p* = 0.022), although the association with OS was not significant. Stage II patients among the G2 group had significantly inferior prognosis both in terms of OS and DFS when compared with those among the G0/G1 group, with survival curves similar to those of Stage III cases.

**Conclusions:**

G-factor based on the expression of p53 and MMP-7 was found to be a promising factor to predict outcome of Stage II/III gastric cancer, and possibly to help select the treatment for Stage II cancer, thus supplementing the conventional TNM system.

## Introduction

To date, staging classification of gastric cancers has been performed by the International Union against Cancer (UICC)/TNM classification system [1] and the general rules of the Japanese classification of gastric carcinoma edited by Japanese Gastric Cancer Association [2]. These stage classifications based on clinicopathological factors are the global gold standard for clinical decision-making. In Japan, the technique and procedure for gastrectomy with lymph node dissection in gastric cancer patients have been established, along with adjuvant postoperative chemotherapy for those who have undergone R0 resection [3]. However, some patients suffer from unexpectedly early recurrence among those who underwent curative surgery even in Stage II cancer, implicating differences in biological characteristics. Tumor biology may not always be reflected in the clinical stage, which represents the extent of cancer spread at the time the disease was diagnosed. Therefore, identification of a new factor that reflects biological characteristics is warranted so as to supplement the clinicopathological factors for more precise prediction of the outcome. The G-Project committee was established by the Japan Society for Gastroenterological Carcinogenesis during their 2005 annual meeting, with an aim of proposing a new TNM-G classification that contains G-factor, a category that evaluates expression of relevant molecules that might influence the outcome. While evaluating the convenience of implementing the TNM-G classification system into daily practice, analysis of expression at the genetic level was considered too challenging. Thus, analysis of protein expression by immunohistological staining became the method of choice for this project. Here, we conducted a multicenter collaborative study to identify an appropriate molecule as a G-factor.

## Patients and methods

### Patient extraction

This study was conducted after obtaining approval from the society’s ethics committee at the annual meeting in 2007, and then requesting approval from the ethics committee of each of the four institutions, Department of Surgical Oncology, Osaka City University graduate School of Medicine (Osaka, Japan); Department of Gastroenterological Surgery, Kanazawa University (Kanazawa, Japan); Department of Surgery and Science, Graduate School of Medicine, Kyushu University (Fukuoka, Japan); and Department of Gastroenterological Surgery, Saga University, Faculty of Medicine (Saga, Japan), that supplied resected specimens. Each institution provided samples according to an implementation planning report. The study protocol conformed to the ethical guidelines of the Declaration of Helsinki, 1975. Recent reports suggest that there was a difference of at least 20 % in 5-year survival when patients were stratified according to the expression of useful prognostic factors [4–11]. To detect as much difference in survival in a retrospective analysis, approximately 100 cases who had recurrence and 100 cases who were recurrence-free after a sufficient follow up were deemed necessary.

The aforementioned four institutions ultimately registered 210 cases of gastric cancer who underwent curative R0 resection by gastrectomy with more than D1 lymph node dissection (D1+ to D2). Of the 210 cases, 104 were Stage II and 106 were Stage III according to the Japanese Classification of Gastric Carcinoma (the 13th edition). In addition, 104 cases were confirmed to have postoperative recurrence or death within 5 years, whereas 106 cases were confirmed to have been recurrence-free for 5–10 years. Upon gaining approval from the ethics committees of each of the aforementioned institutions, tissue samples were obtained, along with clinicopathologic data such as age, gender, occupation, operative procedure, degree of penetration into the wall (pT), lymph node metastasis (pN), final stage, ly and v factors, histological type, presence or absence of adjuvant therapy and regime, recurrence type, postoperative disease-free survival (DFS) and postoperative overall survival (OS). The data were subsequently analyzed by the Department of Oncology at the Institute of Geriatrics and Medical Science, Osaka City University, Graduate School of Medicine.

### Selection of molecules for factor analysis

The G project committee was elected, and as a preliminary step, the members conducted a PubMed literature search of articles published between 1990 and 2005 using the key words “gastric cancer” and “independent prognostic factor.” A total of 822 articles on gastric cancer were extracted and reviewed (Table 1). Of the 50 molecules identified, the molecules or other factors frequently highlighted in both univariate and multivariate analyses as prognostic factors were epidermal growth factor (EGF) (35 papers), p53 (21), vascular endothelial growth factor (VEGF) (18), microsatellite instability (12), TGF-β (6), urokinase-type plasminogen activator (u-PA) (5) and E-cadherin (5). Based on these results and considering the known functions of the molecules, the committee selected p53 and VEGF (VEGF-A and VEGF-C) as candidate molecules to be nominated as G-factor. More recent articles were reviewed in the meantime, and the committee decided to add the following five molecules as candidates, based on their emerging roles in cancer biology reported after 2005: matrix metalloproteinase-7 (MMP-7, a member of the metalloprotease family) [12], human epidermal growth factor receptor 2 (HER2) [13], Regenerating islet-derived family, member 4 (Reg IV) [14, 15], olfactomedin 4 (GW112, an anti-apoptotic factor) [16], and Claudin-18 (the tight junction molecule) [17].

**Table 1:** Paper review of prognostic factors in gastric cancer

Category	Molecules	Number of papers by multivariate (M) or univariate (U) analysis
Oncogene	k-*ras*	
	c-*myc*	
Tumor suppressor gene	*p53*	(M-8^a^, U-13^b^)
	*DCC*	
	*p27*	
	*RUNX3*	
MSI (MMR gene)	MSI/BAT	(M-3, U-3)
Cell proliferation	Cyclin D1	
	PCNA	(M-4)
	MIB-1	
	Ki-67	
Growth factor/cytokine and those receptor	EGF/erbB2	(M-15, U-20)
	VEGF	(M-12, U-6)
	c-met	(M-6)
	TGF-b/Smad	(M-6)
	b-FGF	(U-2)
	PDGF	
	TGF-α	
	IL-6	
	IL-18	
	CCR7	
	TNFR	
Apoptosis signal pathway	Bcl-2	(U-2)
	Bax	
	Caspase 3	
	NF kappa B	
Cell invasion and adhesion	TIMP	(M-4)
	uPA	(M-3, U-2)
	E-cadherin	(M-3, U-2)
	CD4	(U-2)
	MMPs	
	PAI-1, 2	
	AMF	
	KISS-1	
Angiogenesis	Thymidine phosphorylase	(U-4)
Others	COX2	(M-2, U-2)
	Microvessel count	(U-4)
	Metastatic LN ration	

^a^No. of papers by multivariate analysis

^b^No. of papers by univariate analysis. The list of prognostic factors reported in the 822 published articles between 1990 and 2005

### Immunohistochemical staining and evaluation

The surgical specimens were formalin-fixed and paraffin-embedded, and sent to the Department of Molecular Pathology at Hiroshima University (Hiroshima, Japan). Immunohistochemical staining was performed at this facility using eight primary antibodies against the eight candidate factors. The Envision+ staining kit (Dako Corp., Glostrup, Denmark) was used for immunostaining. Paraffin-embedded specimens were sectioned at 4 μm, hydrophilized, and microwaved for 30 min in pH 6.0 citric acid buffer or autoclaved in ethylenediaminetetraacetic acid (EDTA) buffer to activate the antigen. Intrinsic peroxidase was deactivated by incubation with 3 % H_2_O_2_ for 10 min. After rinsing, blocking was performed using sheep serum and reacting with each primary antibody. Anti-p53 primary antibody was cloned name DO-7 (Dako); VEGF-A, polyclonal antibody (Santa Cruz); VEGF-C, polyclonal antibody (American Research Products); Reg IV, polyclonal antibody (see Ref. [14]); GW112, N212 (see Ref. [15]); Claudin-18, polyclonal antibody (Invitrogen); MMP-7, 141-7B2 (Daiichi Fine Chemicals). All primary antibodies were diluted 1:50 and incubated at room temperature for 1 h, washed with phosphate buffered saline (PBS), and incubated with a secondary antibody at room temperature for 1 h. The Envision+ Rabbit Peroxidase Detection System (Dako Corp.) contained the primary rabbit antibodies VEGF-A, VEGF-C, Reg IV, and Claudin-18, whereas the Envision+ Mouse Peroxidase Detection System (Dako Corp.) was used to detect p53, olfactomedin 4, and MMP-7. After washing with PBS, the samples were incubated in diaminobenzidine (DAB) solution for 10 min, stained, washed with PBS, and counter-stained with hematoxylin. Expression levels of p53, VEGF-A, VEGF-C, MMP-7, HER2, Reg IV, olfactomedin 4 and Claudin-18, were analyzed by immunostaining using one representative specimen from the center of the tumor. The stained area was scored by the percentage of immunopositive cells as an index of the expression of each molecule. Cases that were not at all stained were scored as 0, cases with < 10 % of stained tumor cells were 1+, cases with 10–50 % of stained tumor cells were 2+, while cases with > 50 % of stained tumor cells were 3+. Evaluation of immunostaining was conducted independently by two pathologists, and any discrepancies in assessment were discussed and reassessed by microscopy. Scores of 2+ and 3+ were determined as positive for immunostaining.

### Data and statistical analysis

The correlation between a clinicopathological factor and immunostaining result was analyzed by the Chi-square test or Fisher’s exact test. The survival duration was calculated using the Kaplan–Meier method and analyzed by the log-rank test and Cox’ regression analysis to compare the cumulative survival durations in the patient groups. In all tests, a *p* value of <0.05 was considered to represent statistical significance. SPSS statistical software (SPSS Japan, Tokyo, Japan) was used for all analyses.

## Results

### Expression of various molecules as evaluated by immunostaining

**Fig. 1:**
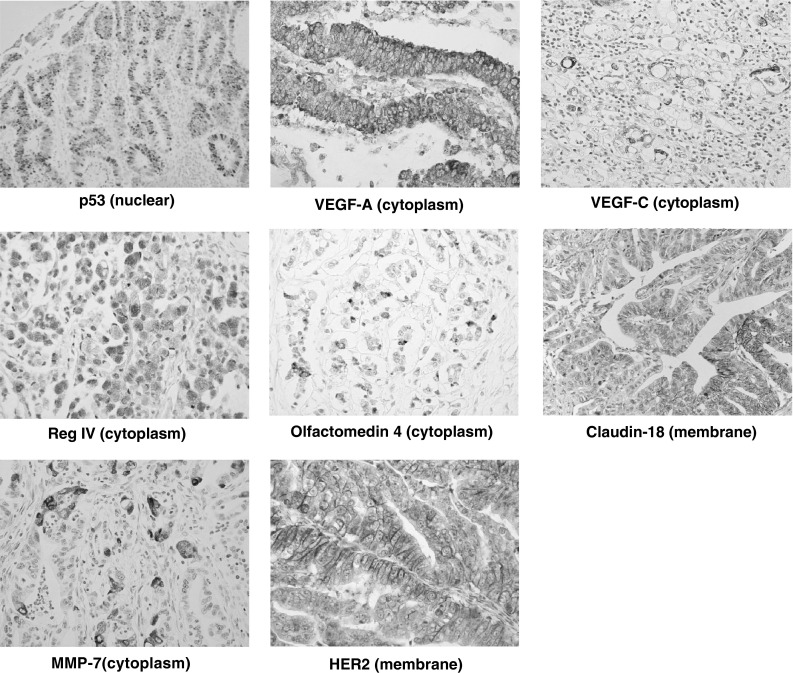
Immunohistochemical findings of p53, VEGF-A, VEGF-C, Reg IV, olfactomedin 4, Claudin-18, MMP7, and HER2

Each representative positive expression in histological image for gastric cancer is depicted in Fig. 1. The positive staining rate of each molecule was 41.4 % for p53, 55.2 % for VEGF-A, 73.3 % for VEGF-C, 25.7 % for Reg IV, 66.2 % for olfactomedin 4, 63.4 % for Claudin-18, 46.7 % for MMP-7, and 13.0 % for HER2, respectively.

### Correlation of postoperative recurrence and clinicopathological factors or the expression of the candidate molecular factors

Examination of the all 210 gastric cancer cases revealed a significant correlation between the postoperative recurrence and pT stage (*p* = 0.008), pN stage (*p* = 0.078), final stage (*p* < 0.001), ly factor (*p* = 0.002), and v factor (*p* < 0.001), but not with surgical procedure or the type of adjuvant chemotherapy received. As for the prognostic significance of candidate molecular factors, no significant correlation was observed between the immunostaining expression of eight aforementioned factors and postoperative recurrence (Table 2).

**Table 2:** Correlation between postoperative recurrence and clinicopathologic features and G-factors expression in 210 patients with gastric cancer

Clinicopathological factors and G-factors expression	Recurrence		*p* value
	Negative *n* = 106 (50 %)	Positive *n* = 104 (50 %)	
Gender			
Male	76 (50 %)	72 (50 %)	1.000
Female	30 (50 %)	32 (50 %)	
Location			
U	24 (41 %)	30 (59 %)	0.290
M	47 (54 %)	42 (46 %)	
L	35 (51 %)	32 (49 %)	
pT			
1	8 (69 %)	1 (31 %)	0.008
2	51 (59 %)	35 (41 %)	
3	47 (43 %)	63 (57 %)	
4	0 (0 %)	5 (100 %)	
pN			
Negative	26 (64 %)	10 (36 %)	0.078
Positive	80 (47 %)	94 (53 %)	
pN			
0	26 (64 %)	10 (36 %)	0.041
1	55 (53 %)	48 (47 %)	
2	24 (36 %)	45 (64 %)	
3	1 (50 %)	1 (50 %)	
Stage			
II	76 (73 %)	28 (27 %)	<0.001
III	30 (28 %)	76 (71 %)	
Histologic type			
Diffuse type	64 (51 %)	62 (49 %)	0.911
Intestinal type	42 (50 %)	42 (50 %)	
Lymphatic invasion			
Negative	32 (69 %)	10 (31 %)	0.002
Positive	74 (45 %)	94 (55 %)	
Venous invasion			
Negative	76 (64 %)	40 (36 %)	<0.001
Positive	30 (32 %)	64 (68 %)	
p53			
Negative	67 (55 %)	56 (45 %)	0.100
Positive	39 (43 %)	48 (57 %)	
VEGF-A			
Negative	55 (56 %)	40 (44 %)	0.105
Positive	51 (45 %)	64 (55 %)	
VEGF-C			
Negative	28 (48 %)	28 (52 %)	0.651
Positive	78 (51 %)	76 (49 %)	
Reg IV			
Negative	76 (49 %)	80 (51 %)	0.536
Positive	30 (54 %)	24 (46 %)	
Olfactomedin 4			
Negative	30 (42 %)	41 (58 %)	0.089
Positive	76 (54 %)	63 (46 %)	
Claudin-18			
Negative	37 (47 %)	39 (53 %)	0.485
Positive	69 (52 %)	45 (48 %)	
MMP-7			
Negative	64 (56 %)	48 (44 %)	0.058
Positive	42 (43 %)	56 (57 %)	
HER2			
Negative	87 (48 %)	96 (52 %)	0.073
Positive	19 (66 %)	8 (34 %)	

### Prognostic analysis of OS and DFS in expression of the candidate molecular factors

**Fig. 2:**
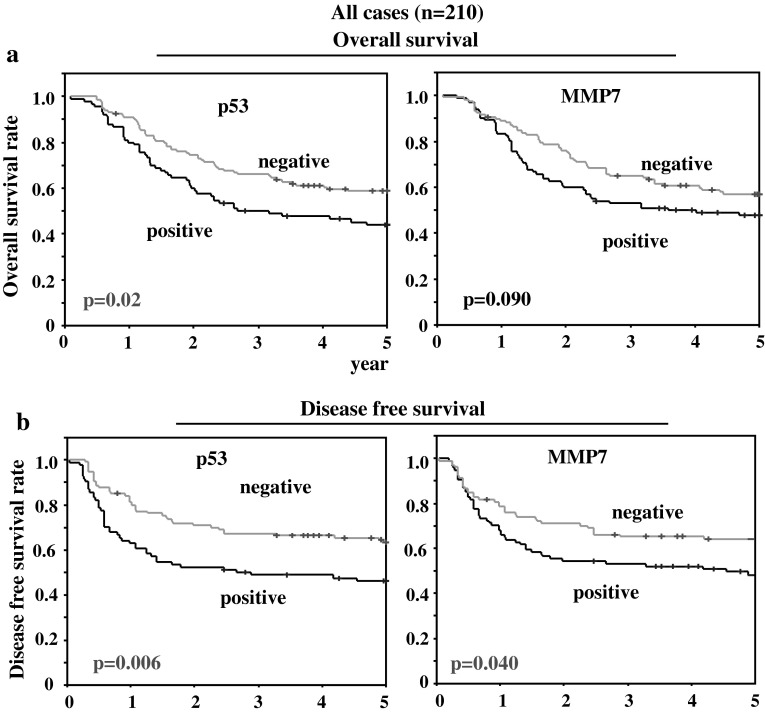
Overall survival curves (**a**) and disease-free survival curves (**b**) of 210 total gastric cancer cases

In terms of OS for all gastric cancer cases (Stage II and III), cases with positive p53 expression had significantly worse prognosis (*p* = 0.02) when compared with p53-negative cases. Cases positive for MMP7 tended to have worse prognosis in comparison with the negative cases, although not significantly so (*p* = 0.09) (Fig. 2a). In terms of DFS, p53-positive and MMP7-positive cases had significantly worse prognosis (*p* = 0.006 and *p* = 0.04, respectively) (Fig. 2b). No significant difference in prognosis was observed between patients positive and negative for four other factors (VEGF-A, Reg IV, olfactomedin 4, and Claudin-18) (data not shown).

**Fig. 3:**
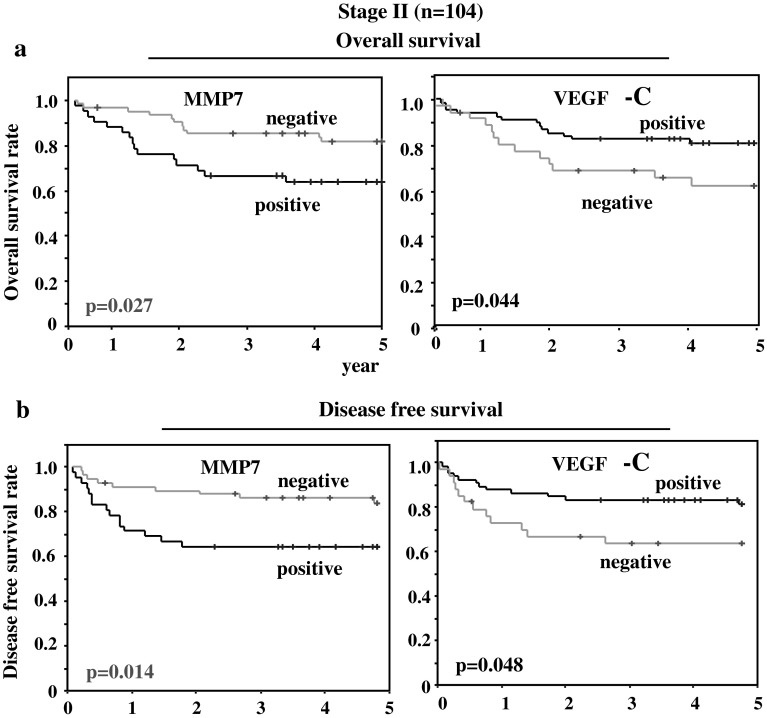
Overall survival curves (**a**) and disease-free survival curves (**b**) of 104 gastric cancer cases at Stage II

Figure 3 reveals prognostic evaluation (OS and DFS) in Stage II gastric cancer cases (*n* = 104). MMP7-positive cases suffered from significantly worse prognosis both in terms of OS (*p* = 0.027) and DFS (*p* = 0.014). In addition, VEGF-C positive cases had more favorable prognosis (*p* = 0.044 for OS and *p* = 0.048 for DFS) compared with VEGF-C negative cases. The similar analysis of OS and DFS revealed no significant difference in survival among Stage III patients (*n* = 106) for any of the eight factors.

### Feasibility of the candidate factors for G-factor

According to the aforementioned results, we selected p53 and MMP-7 as G-factor that might serve our purpose. Consequently, we analyzed the association between the combination of p53 and MMP-7 expression and prognosis. All gastric cancer patients in the current series were stratified into three groups as follows, based on the expression of these molecules; G0 group (negative for both molecules, *n* = 69), G1 group (positive for either of the molecules, *n* = 97), and G2 group (positive for both of the molecules, *n* = 44). In all of the 210 gastric cancer cases, G2 cases demonstrated significantly higher recurrence rate (59 %) compared to 38 % in G0 cases (*p* = 0.047). In stage-by-stage analyses, the recurrence rate of G2 cases (50 %) tended to be higher in comparison with that of G0 and G1 cases for Stage II (25 and 20 %, respectively, *p* = 0.069), but not for Stage III (*p* = 0.414).

In the univariate analysis, G2 cases were associated with a shorter OS (hazard ratio 1.83, 95 % CI 1.15–2.90; *p* = 0.01) and DFS (hazard ratio 2.15, 95 % CI 1.26–3.68; *p* = 0.005) in comparison with G0 and G1 cases. The multivariate Cox’ regression analysis revealed that the G2 group was not an independent prognostic factor for OS (hazard ratio 1.59, 95 % CI 0.99–2.55; *p* = 0.052), but was independently associated with a shorter DFS (hazard ratio 1.90, 95 % CI 1.10–3.30; *p* = 0.022) (Table 3).

**Table 3:** Prognostic factors of overall survival and disease-free survival (univariate and multivariate analyses)

Variable	OS univariate analysis			OS multivariate analysis			DFS univariate analysis			DFS multivariate analysis		
	HR	95 % CI	*p* value	HR	95 % CI	*p* value	HR	95 % CI	*p* value	HR	95 % CI	*p* value
p53/MMP-7 expression												
G2 vs. G0/1	1.83	1.15–2.90	0.010	1.59	0.99–2.55	0.052	2.15	1.26–3.68	0.005	1.90	1.10–3.30	0.022
Depth of invasion												
T1, T2 vs. T3, T4	1.99	1.39–2.87	<0.001	2.40	1.65–3.51	<0.001	1.79	1.20–2.70	0.005	2.17	1.43–3.30	<0.001
Lymph node metastasis												
Positive vs. negative	2.61	1.22–4.82	0.011	3.68	1.75–7.70	0.001	2.86	1.32–6.21	0.008	4.33	1.85–10.15	0.001
Lymphatic invasion												
Positive vs. negative	2.61	1.36–5.03	0.004	1.89	0.95–3.76	0.068	4.41	1.79–10.91	0.001	2.92	1.15–7.41	0.024
Venous invasion												
Positive vs. negative	2.56	1.70–3.85	<0.001	1.90	1.23–2.93	0.004	2.77	1.77–4.34	<0.001	1.94	1.21–3.11	0.06

Cox’s regression analysis; *HR* hazard ratio; *95* *% CI* 95 % confidence interval

**Fig. 4:**
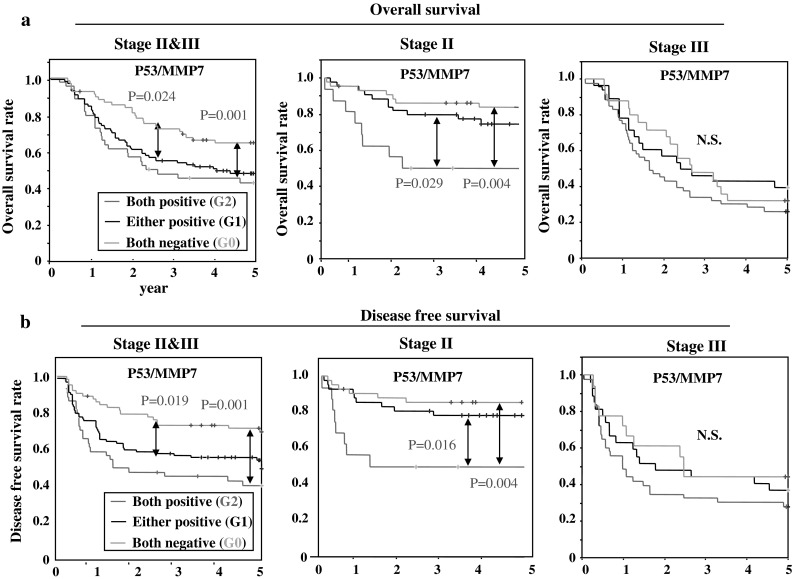
Kaplan–Meier survival curve of patients with gastric cancer in combination with p53 and MMP7 expression (G0–2). Overall survival curves (**a**) and disease-free survival curves (**b**)

Survival analysis revealed that long term outcome of G0 cases was significantly better than that of G1 cases (*p* = 0.024 for OS and 0.019 for DFS) and G2 cases (*p* = 0.001 for OS and DFS) in all cases analyzed. In addition, G2 was particularly strong as marker of poor prognosis among Stage II cancer (*p* = 0.004 for comparison of OS with G0 and 0.029 for comparison with G1) (Fig. 4).

## Discussion

The TNM staging system is widely used throughout the world to predict the prognosis of malignant tumors, in which staging is based on three clinicopathological factors: T factor, the degree of wall penetration of the primary tumor; N factor, the status of lymph node metastasis; and M factor, the presence of distant metastasis [1]. In the case of gastric cancer, 5-year survival rate of Stage I cancer is >90 %, whereas survivors rarely exist among patients with Stage IV cancer. However, the outcome of Stage II or III cases are varied, despite being treated by a similar strategy of radical surgery and adjuvant therapy, reflecting the fact that gastric cancer is a mixed population of cancers with various biologic characteristics that cannot be classified based only on conventional prognostic factors [18–20]. The present study was therefore conducted through the collaboration of several institutions and the Japan Society for Gastroenterological Carcinogenesis, to investigate the feasibility of new molecular staging of a G-factor, which would supplement the conventional TNM staging system.

An extensive literature search was conducted during selection of candidate G-factors, and 50 prognostic factors were identified for gastric cancer, mostly from single institutions. However, no significant correlation with prognosis was observed in most of the molecules thus selected for the current validation set consisting of 210 gastric cancer cases with known prognosis, with the exception of p53. MMP7 was the only other molecule that was of interest, since it was associated with prognosis in the Stage II subset. Moreover, even with p53, the difference in the 5-year survival rate was <20 %. This difference did not meet the criterion generally suggested in resent published reports [4–11, 21–24] that a prognostic difference of ≥20 % is required when predicting the prognosis, based on the biochemical characteristics of cancer, so as to supplement the conventional TNM stage classification. In other words, current study indicated that no single molecule could serve the role of G-factor as originally proposed.

Consequently, we decided to combine p53 and MMP-7 as a new marker of prognosis. Patients were stratified into three groups based on p53 and MMP-7 expression; G0 group (both negative group), G1 group (either positive group), and G2 group (both positive group). Difference in survival between G2 and G0 was shown to be quite significant, and G2 was an independent prognostic factor in terms of DFS and of borderline significance in terms of OS. In addition, a significant difference in OS and DFS was observed between G0/1 and G2 groups among the Stage II cancer subset. Furthermore, the OS and DFS of Stage II G2 cases were similar to those of Stage III cases, and were clearly distinct from other Stage II cancers. New clinical trials for Stage III gastric cancer are either currently ongoing or under preparation based on the fact that hazard ratio for the treatment group incrementally increased as the clinical stage advanced from II to IIIB in a randomized trial comparing adjuvant S-1 monotherapy versus surgery alone [25]. A novel classification with G-factor can now identify a high-risk group among Stage II cancers that may be eligible for these trials and may benefit from more intensive adjuvant treatments.

A number of new molecules or those hitherto unrelated to cancer have been identified in recent researches using various types of arrays. Further efforts to enhance the G2 factor or to find other relevant gene signatures are warranted to improve clinical staging schemes, and to ultimately establish an era of personalized therapy.

## Conclusion

Through a multi-institutional effort led by the Japan Society for Gastroenterological Carcinogenesis, a combination of p53/MMP-7 expression was found to be a promising factor to predict outcome of Stage II/III gastric cancer, and possibly to help select the treatment for Stage II cancer, thus supplementing the conventional TNM system.
